# Response to “Are microsporidia infections really increasing in HIV/AIDS patients or is it increased awareness?”

**DOI:** 10.1093/inthealth/ihag080

**Published:** 2026-08-06

**Authors:** Ali Asghari

**Affiliations:** Medical Microbiology Research Center, Qazvin University of Medical Sciences, Qazvin, Iran

**Dear Editor**,

We thank Dr. Sarman Singh for his interest in our recently published article, “A global overview of microsporidia infection in HIV/AIDS patients: an updated systematic review and meta-analysis”,^1^ and for his Letter to the Editor entitled “Are microsporidia infections really increasing in HIV/AIDS patients or is it increased awareness?”.^2^ We welcome the opportunity to clarify the issues raised and further discuss the interpretation of our findings.

## Rationale for restricting the search period to 2017–2025

Dr. Singh questions the logic of limiting the analysis to studies published from 2017 onward, suggesting that this approach excludes three decades of epidemiological data.

We respectfully clarify that our study was explicitly designed and clearly described (in the title, abstract, introduction, and discussion) as an updated systematic review and meta-analysis. A previous comprehensive meta-analysis by Wang et al., 2018^3^ systematically evaluated studies conducted up to 31 December 2016 and reported a pooled microsporidia prevalence of 11.8% (95% CI: 10.1–13.4%) among HIV/AIDS patients. Our objective was not to duplicate that work but to synthesize evidence published subsequently (1 January 2017 to 11 October 2025), thereby extending the evidence base without overlapping datasets. This issue is explicitly mentioned in the second and third paragraphs of the discussion.^1^

Updated meta-analyses are a well-established and methodologically accepted approach when substantial new evidence accumulates.^4^ Careful assessment confirmed that including studies published before 2017 would have resulted in duplication of primary datasets already synthesized in the previous meta-analysis. A combined analysis was therefore not undertaken to preserve methodological independence and avoid unnecessary overlap.

## Interpretation of post-2020 prevalence patterns

The Letter suggests that our discussion may imply a real increase in microsporidia infections after 2020 and argues that improved molecular diagnostics could explain the observed pattern.

We wish to clarify that our article does not assert a definitive increase in true infection incidence. Rather, we reported that the pooled prevalence in studies published after 2020 (15.9%) was higher than that of studies published before 2020 (8.4%), and explicitly stated that this observation must be interpreted with caution.

Importantly, our discussion already acknowledged multiple plausible explanations, including disruptions in HIV care and ART initiation during the COVID-19 pandemic, laboratory resource reallocation, improvements in molecular diagnostic techniques, and possible epidemiological shifts. For example, the increasing implementation of highly sensitive molecular assays, including nested PCR, real-time PCR, multiplex PCR, and sequence-based species identification, has substantially improved the detection and accurate identification of microsporidia compared with conventional microscopic techniques used more frequently in earlier studies.^5,6^

We explicitly emphasized that the data are limited and exploratory in nature. Thus, the possibility of improved diagnostic capacity contributing to increased detection was already incorporated into our interpretation. Our conclusions do not attribute causality to an epidemiological surge; rather, they highlight a pattern in reported prevalence that warrants further investigation.

## Geographic distribution of studies

The Letter interprets our discussion of the high number of studies from India and Iran as implying surprise or an epidemiological anomaly.

We respectfully clarify that no such implication was made. Our discussion noted that certain countries contributed a disproportionate number of studies, whereas many regions were represented by only one or two investigations. This imbalance affects the generalizability of the pooled global estimate.

Our point concerned representational weighting and interpretative caution, not bibliometric surprise nor geopolitical inference. Highlighting unequal study distribution is standard practice in meta-analytic reporting and directly relates to external validity.

## Pediatric versus adult stratification

It was suggested that Table 1 should have distinguished between pediatric and adult populations. We would like to clarify that subgroup analyses in our study were restricted to variables that were consistently reported across multiple studies with sufficiently detailed and extractable data. However, across the included studies, complete and comparable reporting of subgroup-specific denominators (i.e., the total number of individuals within each category along with the corresponding number of microsporidia-positive cases) was frequently lacking.

**Table 1:** Distribution of positive microsporidia cases in HIV/AIDS patients, categorised by age, sex, CD4 T cell count, and diarrhea.

Variables	Prevalence % (95% CI) ^^a^^	No. datasets ^^b^^	df (Q)	Heterogeneity (Q)	I^2^
**Age groups (Y)** ≤40 >40	55.9 (20.8-86) 44.1 (14-79.2)	2 2	1 1	3.6 3.6	72.5 72.5
**Sex** Female Male	35.1 (20.9-52.5) 64.9 (47.5-79.1)	4 4	3 3	2.4 2.4	0 0
**CD4 T cell count** ≤200 >200	58.4 (46.4-69.4) 36.9 (25.3-50.2)	17 15	16 14	38.1 35.9	57.9 61.1
**Symptoms** Diarrhea Non-diarrhea	36.4 (27.8-45.9) 61.9 (49.7-72.8)	7 5	6 4	6.1 5.8	1.5 31.2

a The reported values represent the proportion of microsporidia-positive cases within each category (age, sex, CD4 count, and diarrhea) relative to the total number of positive cases, rather than true subgroup-specific prevalence. This is because the denominators (i.e., the total number of individuals tested in each group) were not consistently provided across studies.

b The variation in the number of datasets arises from the fact that not all included studies reported data for every classification within a given variable. In some studies, for instance, all HIV/AIDS participants or all microsporidia-positive cases belonged to a single category (e.g., only ≤40 years or >40 years). Such inconsistencies limited the ability to calculate accurate proportions. Therefore, these findings should be interpreted with caution, as they primarily reflect the distribution of available data rather than definitive epidemiological patterns.

As a result, a formal subgroup meta-analysis estimating true prevalence across age categories was not feasible. Instead, the data presented in Table 1 reflect the proportional distribution of microsporidia-positive cases across available categories, rather than subgroup-specific prevalence estimates. This distinction was explicitly stated in the manuscript to avoid misinterpretation.

Furthermore, the limited number of studies evaluating age-specific patterns, along with heterogeneity in age categorization, precluded a meaningful and statistically robust comparison across age groups, including between pediatric and adult populations.

This limitation highlights the need for future studies with standardized and detailed reporting of age-stratified data. In particular, broader inclusion of diverse age groups and more consistent subgroup definitions will be essential to enable comprehensive and reliable age-specific analyses of microsporidia infection in HIV/AIDS patients.

## Conclusions regarding infection trends

Finally, we reiterate that the overall pooled prevalence of microsporidia infection among HIV/AIDS patients in our study was 12% (95% CI: 8.9–16%), which is highly comparable to the 11.8% (95% CI: 10.1–13.4%) reported in the pre-2017 meta-analysis. In fact, our discussion explicitly states that this slight difference suggests a relatively stable burden over time despite expanded geographic coverage and evolving methodologies. At no point did we claim a definitive global increase in microsporidia infections. Our wording consistently reflects caution, limitation of available data, and the need for further surveillance.

## Concluding remarks

We appreciate the opportunity to further clarify several aspects of our methodology and interpretation. We hope that this response provides additional context for readers and contributes to continued scientific discussion regarding the epidemiology of microsporidia infection in HIV/AIDS populations.

## Data Availability

Data are available in the article and references.
